# Editorial: A Systems View of Plant Cellular Communication

**DOI:** 10.3389/fpls.2022.875046

**Published:** 2022-03-21

**Authors:** Dario Di Silvestre, Luca Tadini, Andrea Trotta, Luis Valledor, Ghasem Hosseini Salekdeh, Jesus V. Jorrin Novo

**Affiliations:** ^1^Institute of Biomedical Technologies, National Research Council, Segrate, Italy; ^2^Department of Biosciences, University of Milan, Milan, Italy; ^3^Institute of Biosciences and Bioresources, National Research Council, Sesto Fiorentino, Italy; ^4^Department of Organisms and Systems Biology, Institute of Biotechnology of Asturias, University of Oviedo, Oviedo, Spain; ^5^Agricultural Biotechnology Research Institute of Iran, Karaj, Iran; ^6^Department of Biochemistry and Molecular Biology, ETSIAM, University of Cordoba, Córdoba, Spain

**Keywords:** systems biology, plant communication, OMICS data, network analysis, modeling

This special issue (SI) provides a holistic view of plant cellular communication at the basis of plant development and stress responses. Since inter- and intra-cellular cellular communication pathways comprise complex molecular interactions, this SI also aimed to highlight research contributions integrating multiple -omics data and network models. Studies included in this collection covered many topics, ranging from plant-pathogen interaction (Ferreira-Neto et al.; Sekiya et al.) to organs (Castañeda et al.), cells (Thibivilliers and Libault), and organelles (O'Conners and Li) communication. Other studies provided a system perspective on molecular relationships underlying seed development (Cloutier et al.) and prion-like proteins (Garai et al.).

In the context of plant-pathogen interaction, Sekiya et al. contributed with a study that provides insights on the early response of *Eucalyptus grandis* to *Austropuccinia psidii* infection. Cellular communication was addressed by integrating proteomics and metabolomics data in co-expression network modules (WGCNA). Moreover, epifluorescence microscopy was used to follow the fungus development inside the leaves of rust-resistant and -susceptible plants. The results allowed determining comparative spatial and temporal differences in metabolites and proteins. Specifically, 16 metabolites of the phenolic chemical group were found to be involved in resistance and susceptibility to rust. Thus, by this study, the authors provided putative targets for breeding efforts and new strategies for enhancing plant disease resistance.

A different strategy in investigating plant-pathogen relationship was adopted by Ferreira-Neto et al. They focused on cowpea protein kinases (PKs or “kinome”) by evaluating their structural characteristics (at genomic and proteomic level), evolutionary aspects, conservation among *Viridiplantae* species, and gene expression in three cowpea genotypes under biotic (CABMV or CPSMV) and abiotic (root dehydration) stresses. Structural analysis revealed the presence of 1,293 PKs distributed in 20 groups and 118 families, while transcriptomic analysis suggested a stress-, genotype-, or tissue-dependent PKs regulation. The authors highlighted that regulation of PKs under root dehydration induce defense mechanisms against biotic stress, too. Moreover, the study confirms the presence of universal defense mechanisms, and provides interesting starting points for further investigation on the evolution and molecular function of PKs in stress responses.

The role of cellular communication upon drought stress was investigated by Castaneda et al. The aim was to understand long-distance signaling, between both cells and organs. Rather than gene expression, the authors focused on shotgun proteomics which can reveal the actual localization and dynamics of proteins and networks. Starting from the question of whether protein isoforms may exist ubiquitously across organs, a core stress responsive proteome (CSRP) counting 92 protein isoforms was found to be shared ubiquitously amongst several *Medicago truncatula* tissues, including roots, phloem sap, petioles, and leaves. Most of them, significantly involved in drought stress, redox homeostasis and signaling, were connected through a protein–protein interaction network confirming a global role of CSRP in a long-distance stress-responses. The authors brought out the function of the phloem, where the expression level of CSRP was more abundant than other tissues. These findings suggested a new role of the phloem in coordinating stress response, by enabling communication between cells, organs and environment. However, since cell-to-cell and organs communication research is still in its infancy, further efforts and studies are required to improve knowledge on the field, also looking to other potentially key components, like extracellular vesicles, still poorly investigated in plants.

To give a perspective on plant single-cell biological datasets collection and analysis in the context of cellular interactions, Thibivilliers and Libault provided a point of view on the challenges and strategies to collect plant single-cell datasets. This study takes place in the context of cellular interactions controlling the dynamic response of a plant, to abiotic or biotic stresses, as well as in relation to developmental stages. As an example, the authors used transcriptomic data on *Arabidopsis thaliana* genes controlling the differentiation of the root hair cells. This strategy, spatially applied, offered the opportunity to access the transcriptome of cells in the context of the organ morphology. On the other hand, the knowledge accumulated by single-cell omics technologies can also be exploited to enhance the functional annotation of plant species with limited annotations in this field. However, spatial omics technologies are currently almost exclusively restricted to transcriptomic analysis, while single-cell proteomics is still in an early stage. Nevertheless, overcoming some limitations typical of plant organisms, they will represent the technological advances to capture the entire diversity and subtle differences existing between cells, to reveal cell-type-specific regulatory networks and biological processes.

An interesting contribution by O'Conners and Li focused on organelle communication. They described a process of intracellular genome crosstalk called “Mitochondrial Fostering,” where the plant mitochondrial genome plays a role in developing *de novo* orphan genes, as a continuation of gene transfer that began with endosymbiosis. Following a comparison between mammal and plant genomes, the authors found that mammal genomes are much larger, while the mitochondrial ones are much smaller than in plants, where they are also much more dynamic in size between different species. Since plants also showed more nuclear mitochondrial DNA (NUMT), the authors suggested that plants mitochondrial genomes play an active role in orphan gene evolution more than in mammals. In addition to providing information on evolutionary theory, the functionality associated with orphan genes may be of great interest. In fact, they are species-specific and their role in molecular networks represents a topic which deserves future investigation.

As for integrative modeling, Cloutier et al. investigated the metabolic network driving the compound accumulation in *Arabidopsis* seeds. The authors applied an Ordinary Differential Equations model and, through *in silico* perturbation of genes, they found targets correlated with seed oil traits. These findings were validated by scientific literature and by integrating time-course gene expression profiling during *Arabidopsis thaliana* embryo development. The identification of genes correlated with seed oil traits, as well as the use of integrative approaches, may conduct metabolic engineering, drug design and synthetic biology. However, studies are suggesting that storage in plant seeds is affected by multiple interacting pathways and molecular networks, thus to guide metabolic engineering it will be fundamental to develop more and more tools to unveil the complexity of the systems and their emergent properties.

Again, in the scenario of integrative modeling, Garai et al. pointed their attention to prion-like proteins (PrLPs) defining the so called “prionome.” PrLPs are a subclass of amyloid proteins which in their aggregated state can act as heritable elements over generations. Since prion-like behavior has been correlated with “long-term memory,” the authors hypothesized that plant PrLPs may also possess a potential role in stress and memory. However, in addition to beneficial aspects, they could induce harmful effects like in the case of protein inactivation, toxic function, or impaired homeostatic physiological functions. By integrating the available rice interactome, transcriptome and regulome data sets, the authors found links between stress and memory pathways. By this integrative meta-analysis, they connected PrLPs with transient and transgenerational memory mechanisms, suggesting that plant memory may rely upon protein-based signals in addition to chromatin-based epigenetic signals. Of note, several PrLPs were predicted to have a multi-subcellular localization, mainly mitochondrial, chloroplastic and cytosolic, suggesting also a role in transmitting signals from the organelle to the nucleus under stress conditions. Moreover, some PrLPs were defined as “hubs” in the rice co-expression networks, suggesting a role of key mediators of stress and memory connections, thereby helping plants to fortify defenses for a stronger and rapid response by retaining memories of the last event.

In conclusion, this special issue highlighted how integrated approaches could represent a successful strategy in shedding light on complex molecular mechanisms underlying cellular communication. Through the contributions included therein, the interest and importance in studying plants of industrial or commercial interest emerged, for improving their defense systems and, therefore, the production processes. In addition to new concepts oriented to systems biology, such as “prionome” or “kinome,” it has been interesting to observe how new technologies, i.e., single cells, are increasingly used also by the plant research community. In the same way, the study of aspects that can find analogies or divergences with the animal world appeared equally relevant, as in the case of prion proteins or the transfer of genome from mitochondria to the nucleus.

## Author Contributions

All authors listed have made a substantial, direct, and intellectual contribution to the work and approved it for publication.

## Conflict of Interest

The authors declare that the research was conducted in the absence of any commercial or financial relationships that could be construed as a potential conflict of interest.

## Publisher's Note

All claims expressed in this article are solely those of the authors and do not necessarily represent those of their affiliated organizations, or those of the publisher, the editors and the reviewers. Any product that may be evaluated in this article, or claim that may be made by its manufacturer, is not guaranteed or endorsed by the publisher.

